# A Soft Sensor Approach Based on an Echo State Network Optimized by Improved Genetic Algorithm

**DOI:** 10.3390/s20175000

**Published:** 2020-09-03

**Authors:** Ruoyu Huang, Zetao Li, Bin Cao

**Affiliations:** 1 The Electrical Engineering College, Guizhou University, Guiyang 550025, China; ruoyuhuang@126.com; 2 Guiyang Aluminum Magnesium Design and Research Institute Co., Ltd., Guiyang 550081, China; 3 Chinalco Intelligent Technology Development Co., Ltd., Hangzhou 311199, China; caobinh@yeah.net

**Keywords:** soft sensor, echo state network (ESN), genetic algorithm (GA), alumina concentration, aluminum reduction cell

## Abstract

In the process of fault diagnosis and the health and safety operation evaluation of modern industrial processes, it is crucial to measure important state variables, which cannot be directly detected due to limitations of economy, technology, environment and space. Therefore, this paper proposes a data-driven soft sensor approach based on an echo state network (ESN) optimized by an improved genetic algorithm (IGA). Firstly, with an ESN, a data-driven model (DDM) between secondary variables and dominant variables is established. Secondly, in order to improve the prediction performance, the IGA is utilized to optimize the parameters of the ESN. Then, the immigration strategy is introduced and the crossover and mutation operators are changed adaptively to improve the convergence speed of the algorithm and address the problem that the algorithm falls into the local optimum. Finally, a soft sensor model of an ESN optimized by an IGA is established (IGA-ESN), and the advantages and performance of the proposed method are verified by estimating the alumina concentration in an aluminum reduction cell. The experimental results illustrated that the proposed method is efficient, and the error was significantly reduced compared with the traditional algorithm.

## 1. Introduction

For industrial enterprises, energy-saving, cost reduction and efficiency increase are the foundation of their development. The main means are to continuously optimize the control strategy of the production process and enhance the state monitoring of production equipment and process. One of the problems to be solved is to collect the key state variables in real time. However, these variables are usually difficult to measure directly. The main reason is that on the one hand, they are limited by the harsh environment and narrow space. There is no suitable hardware sensor to adapt to special industrial sites. On the other hand, the development of available hardware sensors cannot be realized in a short period of time in terms of technology and economy. In addition, generally speaking, modern industry can be regarded as a complex nonlinear system, and large uncertainties exist in the processes. For example, the sampling time of some process variables is irregular, since they may have been tested in the laboratory, which could result in varying time delay. Even if some state variables can be measured directly, the measured data may be extremely unreliable [1,2,3,4,5,6].

**Figure 1:**
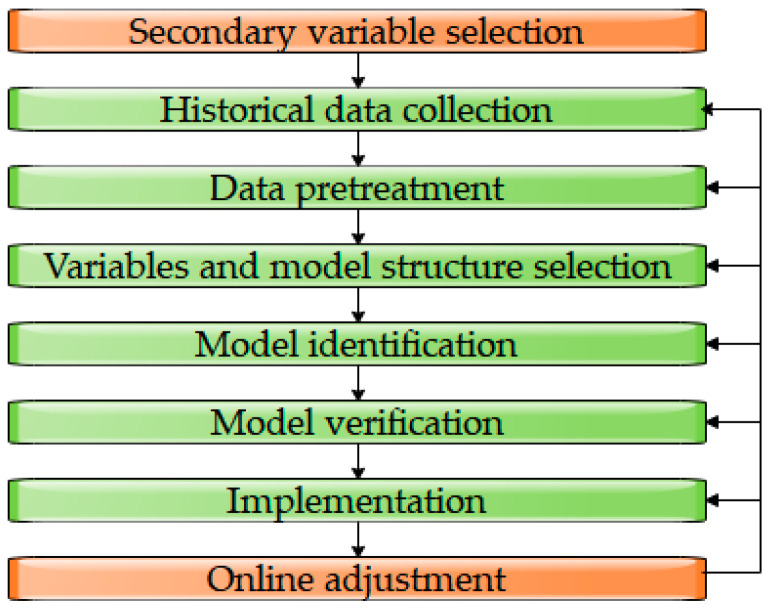
The typical steps of soft sensor development.

One way to solve the above issue is to develop a soft sensor, which seeks to select one or more variables that are easy to measure as secondary variables, and estimate the target variables by establishing a mathematical model according to the correlation between the selected variables and the target variables [7,8,9]. Generally, developing a reliable soft sensor consists of the following steps (see Figure 1): secondary variables selection, historical data collection, data pretreatment, variables and model structure selection, model identification, model verification, implementing and online adjustment. In the adjustment process, steps 2–6 will be repeated until a certain estimation accuracy is achieved [10].

Among them, the variables and model structure selection is a relatively critical step, which needs to determine an appropriate and reliable soft sensor model according to the actual requirements. In general, soft sensor models can be divided into two categories: first principles models (FPMs) and data-driven models (DDMs). As described in Referrence [7], the methods of FPMs are based on physical and chemical principles and assumes the process as an ideal static state. However, in many industrial processes, there are some characteristics, such as unclear mechanisms, multi-physical coupling, object–model mismatch and nonlinearity, which make it difficult to realize ideal physical assumptions and deviation measurements, which in turn limits the application of such methods. By contrast, developing a soft sensor based on DDMs only requires obtaining the historical operational data without considering the complex mechanism or process knowledge, and this has been successfully applied to many spheres, which has attracted more and more attention from academia and industry. Classical data-driven modeling methods include principle component analysis (PCA) [11,12,13,14,15,16], support vector machine (SVM) [17,18,19,20], partial least squares (PLS) [21,22], Gaussian process regression (GPR) [23,24], Bayesian prediction [25,26], slow feature analysis (SFA) [27,28,29,30], extreme learning machine (ELM) [31] and their improved models, artificial neural networks (ANNs) [32] and two or more hybrid models [33,34,35], among others. The advantage of PCA is that it is convenient for simplifying the model and is generally used for the correlation analysis between the same matrix vectors. PLS is used to simulate the correlation between independent variables and dependent variables, which are commonly used in linear systems. SVM is generally used for the binary classification of vectors in the feature space. GPR needs to use a complete sample or characteristic information to predict, and losses effectiveness in high dimensional space. Bayesian prediction needs to know the prior probability and is sensitive to the expression of input data. SFA performs well in analyzing the invariant characteristics of time-varying signals, capturing the dynamic changes of process variables and improving the prediction performance.

Comparatively, ANN has strong nonlinear and adaptive information processing capacities. In recent years, the research on ANN has been continuously deepened, and many widely applied network structures have emerged, ESN being one of them. ESN is a new architecture of recurrent neural network (RNN), which was proposed by Jaeger et al. at the beginning of this century [36,37]. Because of its excellent performance in nonlinear dynamic system modeling, especially as a “black box” time series model, ESN has been successfully applied in speech recognition, network communication and other fields and achieved good results [38,39,40].

In brief, the above data-driven modeling methods have their own advantages and disadvantages, and due to the limitations of process data, the prediction accuracy can only be guaranteed in the local range. In addition, various industrial processes are multivariable, are time-varying with dynamic delay and have a wide operation range, which make it difficult to achieve satisfactory results. A new data-driven soft sensor, called an improved genetic algorithm (IGA) optimization ESN model (IGA-ESN) is proposed in this paper, inspired by the idea of the abovementioned methods, using the advantages of the ESN in “black box” time series modeling (for example, an aluminum electrolytic cell can be regarded as a non-linear time series black box system), and the ESN’s advantages that can alleviate local minimum and fast learning speed. It then uses IGA to optimize the reservoir parameters of the ESN to raise prediction performance. Then, we take alumina concentration in the aluminum reduction process as a case study to verify the effectiveness of the proposed approach.

It should be noted that many scholars have tried to optimize the ESN by using the IGA and achieved good prediction results. As in reference [41], this method is used to predict the network delay. Compared with these methods and applications, in addition to improving the crossover and mutation operations of the traditional genetic algorithm, in order to avoid the risk that the population diversity will be weakened and the algorithm will fall into the local optimal solution, an immigration strategy is introduced to improve the search ability of the algorithm.

Specifically, several key contributions of this paper can be summarized as follows: (1) Task oriented: as the key variables of industrial systems are difficult to measure in a direct and timely manner, we propose a new data-driven soft sensor, named the IGA-ESN model, which is established by ESN; the reservoir parameters are optimized by IGA to maximize the estimation accuracy and improve the convergence speed. (2) Compared with the traditional data-driven statistical approach, the proposed soft sensor approach significantly improves the convergence speed. In addition, it is an unsupervised learning method, which only needs a small amount of training data. Therefore, the proposed method is even more adaptive for the modern industrial requirement. (3) The established model in this paper is applicable to estimate alumina concentration in an aluminum reduction cell. To the best of our knowledge, this is the first utilization of an ESN model in this field.

The remaining of this paper is structured as follows: In Section 2, the basic characteristics of the ESN, such as reservoir parameters, are introduced. The proposed IGA-ESN model is described at length in Section 3. In Section 4, the experiments and results analysis are presented. Finally, our work is briefly summarized in Section 5.

## 2. ESN Characterization

**Figure 2:**
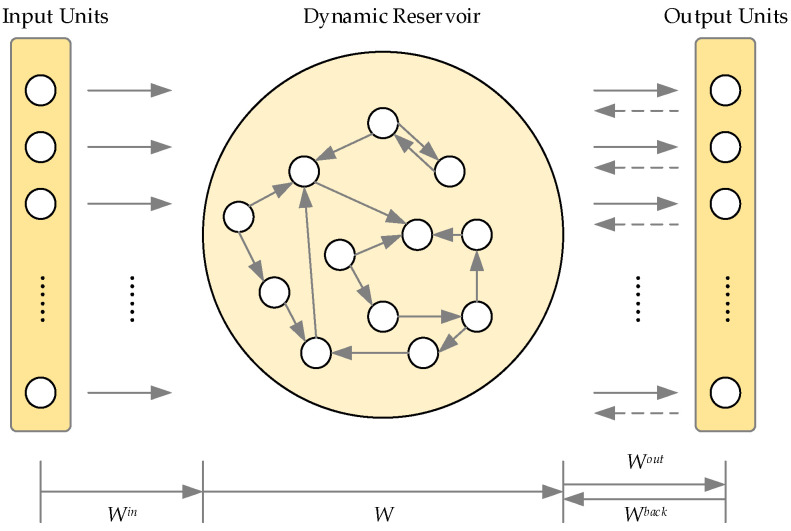
Typical echo state network structure.

A typical structure of an ESN shown in Figure 2. It mainly includes three parts: input units, a dynamic reservoir, and output units. Let the number of nodes in the input and output layers of the ESN be m and l, respectively, and the number of neurons in the reservoir be n. The values of the three parts at time k are u(k), x(k) and y(k), respectively. It has the following expression:

(1) $$u\left(k\right)={\left[u_{1}\left(k\right),u_{2}\left(k\right)\cdots ,u_{m}\left(k\right)\right]}^{T},\;\;x\left(k\right)={\left[x_{1}\left(k\right),x_{2}\left(k\right)\cdots ,x_{n}\left(k\right)\right]}^{T},\;y\left(k\right)={\left[y_{1}\left(k\right),y_{2}\left(k\right)\cdots ,y_{l}\left(k\right)\right]}^{T}$$

The dynamic reservoir is the most important part of the network, which is formed by the sparse connection of many networks. The internal vector of the reservoir is updated in real-time during the network operation. The update equation is as follows:

(2) $$x\left(k+1\right)=f\left(W^{in}u\left(k\right)+W^{res}x\left(k\right)+W^{back}y\left(k\right)\right),$$

where $f=\left[f_{1},f_{2}\cdots ,f_{n}\right]$ is the activation function of the neurons inside the network. $W^{in}$ is the input connection weight matrix and it achieves the connection between the input signal and the reservoir. $W^{res}$ is the reservoir matrix. The network output results will be powered back to the network. The feedback connection is defined as $W^{back}$ in this paper. $W^{back}$ is the connection between the output and the reservoir. The output is connected to the reservoir through $W^{out}$.

The parameter selection of the reservoir is very significant to the performance of the ESN, and it is one of the important problems to be studied in ESN [42]:

- Spectral radius (SR)

The internal connection spectral radius of the reservoir refers to the maximum absolute value of the eigenvalue of $W^{res}$, which is used to represent λ_max_. When λ_max_ *<* 1, the network can be guaranteed to reach a stable state after some time. This is because if λ_max_ *>* 1, the input matrix may come from an empty set, and then the network will enter an instable null state and two stable states, thus violating the echo state property. For a specific proof please see reference [36].

- Processing unit (N)

The number of processing units in the reservoir is the scale of the reservoir, which refers to the number of neurons in the reservoir. When the unknown system is quite complicated, the number of N should be extended. However, the hidden danger is that if N is too large, the network is prone to over-fitting. Over-fitting makes the network achieve a good fitting effect on the training set, while a large deviation appears on the validation set, resulting in a decrease in the prediction performance of the network.

- Input scaling (IS)

Input scaling of the reservoir refers to transforming the input data. When entering data, the data are usually not directly input into the network but scaled to achieve the signal transformation. Empirically, the stronger the non-linear relationship of the tasks in general, the stronger the IS’s expansion force.

- Sparseness of reservoir (SD)

The sparseness of the internal connection of the reservoir is represented by SD. The larger the SD value, the tighter the connection between the networks, the stronger the ability to express information, and the stronger the non-linear approximation ability. However, during the operation of the algorithm, the network calculation amount will increase and the real-time performance will decrease. Choosing the right SD is also crucial.

## 3. Development of the ESN Soft Sensor Model

To achieve a reliable estimation of industrial process state variables that are difficult to measure directly, three points need to be paid attention to. First of all, the historical data sets are highly nonlinear and change dynamically with time, and it is difficult to achieve effective estimation using general statistical methods. Second, the small sample size of the sampling and assay data is not conducive to the use of a large number of data training methods. Third, due to the complex industrial environment, data will inevitably be missing. In order to solve these problems, the data-driven soft measurement method proposed in this paper is mainly based on an ESN suitable for small sample sizes and nonlinear dynamic “black-box” time series modeling. Considering that the parameter selection of reservoir affects the performance of ESN, an improved genetic algorithm was adopted to optimize the parameters of the reservoir. In the process of data acquisition, cubic spline interpolation was adopted to solve the problem of missing data.

### 3.1. Basic ESN

The reservoir state equation and output layer equation obtained from input and output is respectively expressed as:

(3) $$\begin{array}{l} x\left(k+1\right)=f\left(W^{in}u\left(k+1\right)+W^{res}x\left(k\right)+W^{back}y\left(k\right)\right)\\ y\left(k+1\right)=W^{out}x\left(k+1\right) \end{array}$$

In practice, the activation function of neurons is a generally hyperbolic tangent function described in Equation (3). The key issue of ESN is to identify the output connection weight W^out^ using known samples [43]. Suppose the time-series input and output samples are expressed as (u(1),y(1),⋯u(k),y(k)), where u and y are dimension m and dimension l vector, respectively. The samples have a one-to-one correspondence, and this correspondence is what ESN needs to learn. Learning is divided into two phases: the sampling phase and the weight calculation phase.

In the sampling phase, the initial state of the network is arbitrarily selected. Generally, the initial state of the network is chosen as x(0) = 0. The training sample u(k),(k=1,2,⋯m) completes the system state calculation and the corresponding $\hat{y}\left(k\right)$ calculation according to Equation (3) Each time x(k) is calculated. The sample data need to be written to the output units. In practice, to remove the impact of any initial state on the dynamic performance of the system, the state of the system is always collected from a certain moment t_0_. From time t_0_, the system network expresses the mapping relationship between the input and output sample data sets. We define state matrix as B and the output matrix as Q. When $t>t_{0}$, start to collect the corresponding internal state x(k) of the network, add it to the matrix B and finally get the size of the matrix $\left(t-t_{0}+1\right)*n$. After t_0_, the expected output is collected and updated into the matrix Q, and the size of the final Q is $\left(t-t_{0}+1\right)*l$.

In the previous step, the system state matrix B and output matrix Q were obtained. Based on these two matrices, the output connection weight $W^{out}$ is calculated. The relationship between the state variable and the output connection weight is linear. Therefore, the goal to be achieved is to approximate the actual output y(k) according to the output $\hat{y}\left(k\right)$ of the network, which is reflected in the following equation.

(4) $$y\left(k\right)\approx \hat{y}\left(k\right)=\sum_{l=1}^{l}{W_{i}}^{out}x_{i}\left(k\right)$$

It is hoped that the mean-square error of the system satisfying $W_{i}{}^{out}$ is calculated to be the smallest, that is, to solve the optimization problem shown by the following equation:

(5) $$\min\frac{1}{t-t_{0}+1}\sum_{k=t_{0}}^{t}{\left(y\left(k\right)-\sum_{i=1}^{l}W_{i}{}^{out}x_{i}\left(k\right)\right)}^{2}$$

This is a linear regression issue [44]. The output weight can be calculated by solving the inverse matrix:

(6) $${\left(W^{out}\right)}^{T}=B^{-1}Q$$

**Figure 3:**
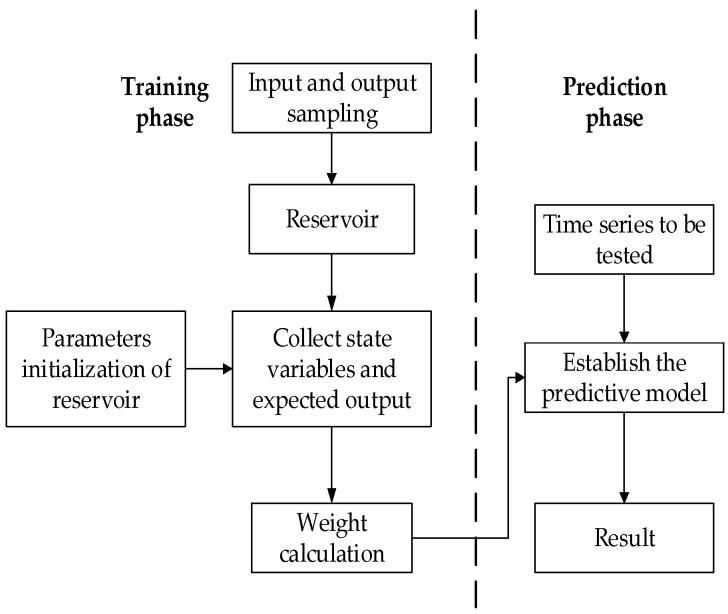
The predictive process of echo state network (ESN).

The inverse matrix B is usually replaced by a pseudo-inverse B, so that all parameters of the ESN can be determined, and the training of the network is completed. Figure 3 is a block diagram of ESN.

### 3.2. IGA-ESN

As outlined in the above section, ESN performance is affected by reservoir parameters and needs to be optimized. A genetic algorithm has the following advantages: first, it takes the value of the objective function as the search information directly, and thus it does not need to differentiate the objective function, avoiding the situation that many objective functions are difficult to differentiate in reality or even of there being no derivative. Secondly, its swarm search characteristics can effectively avoid the problem of searching for some unnecessary points and falling into the local extremum. Therefore, in the established ESN model, an improved genetic algorithm is adopted in order to optimize the reservoir parameters.

#### 3.2.1. Individual Coding

Coding is the first problem to be solved in the application of a genetic algorithm, and it is also a key step in the design of genetic algorithms. The coding method affects the operation methods of the crossover operator, mutation operator and other genetic operators, and largely determines the efficiency of the genetic evolution. Therefore, the parameters of ESN to be optimized are coded first. The binary code operation is simple and easy, and crossover, mutation and other genetic operations are easy to implement; thus this paper uses the binary coding method to code these parameters.

**Figure 4:**
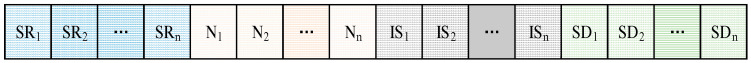
The individual parameter structure of the echo state network (ESN).

The parameters of the ESN to be optimized are: SR, N, IS and SD. We set the genetic algorithm (GA) parameters with a standard deviation of iteration less than or equal to 0.06, and set the number of iterations at 300 times. The individual binary coding takes the following form (See Figure 4).

Individual binary strings encoded by individual parameters are converted to the model’s decimal number by the equation below to represent the actual parameters of the model:

(7) $$X=\min X+\frac{\max X-\min X}{2^{j}-1}d$$

where X is the actual decimal value, minX is the minimum value of the actual decimal number, maxX is the maximum value, j is the length of parameter binary string and d is the decimal number corresponding to the encoded binary string. When setting the coding interval of the reservoir parameters, it is necessary to consider the requirements of ESN training to improve training efficiency. To ensure the network has an echo function, the spectral radius SR was set between [0.1, 0.99], the number of binary code bits was set to 7 bits, the scale of IS was set to [0.01, 1] and the binary code was set to 7 bits. The scale of reservoir N was set to [50, 300], the binary was 9 bits, the reservoir sparseness SD was set to [0.1, 1], the binary code was set to 7 bits, the binary code was 30 bits in total and the population size was designed to be 50. It randomly generated $n_{1},n_{2}\cdots ,n_{50},$ numbered sequentially.

#### 3.2.2. Fitness Function Design

The optimization goal of the genetic algorithm is to find suitable SR, N, IS and SD, and the fitness function should be related to the accuracy of the prediction model. In this paper, the fitness function for the i-th iteration is defined as the value of 1 minus the root mean square error between the actual value and the predicted value of the variable to be measured. If there are several populations, there will be a corresponding number of fitness values. The formula is as follows:

(8) $$Fitness\left(i\right)=1-RMSE\left(i\right)=1-\sqrt{\frac{\sum_{t=1}^{L}{\left(c_{k}-{\hat{c}}_{k}\right)}^{2}}{L}},\left(i=1,2,\cdots ,50\right)$$

where L is the actual length of the predicted sequence, and $c_{k}$ and ${\hat{c}}_{k}$ represent the actual and predicted values of the variable to be measured at time t, respectively. The fitness function of the chromosome can be then be calculated on the basis of the conditions in Formula (8). Each time, 50 fitness values can be obtained using the fitness function to calculate the strengths and weaknesses of each individual.

#### 3.2.3. Selection Operation

The larger the fitness value, the more accurate the model prediction, the better the parameter selection, and the smaller the error. In this paper, the fitness values are added together to obtain a value q, which is defined as the total fitness value:

(9) $$q=\sum_{i=1}^{50}Fitness\left(i\right)$$

The corresponding individual selection probability can be obtained by dividing each fitness value by the total fitness value, and the formula for individual selection probability p(i) is as follows:

(10) $$p\left(i\right)=\frac{Fitness\left(i\right)}{q}$$

The cumulative probability m(i) can be obtained through the cumulative addition of individual selection probabilities. The cumulative probability is divided into 50 intervals, and 50 random numbers r are generated between [0, 1].. If r<s(1), individual $n_{1}$ would be selected to enter the next generation; otherwise, individual $n_{i}$ would be selected to enter the next generation, where m(i−1)<r<m(i) holds, and the selection operation is completed.

#### 3.2.4. Crossover and Mutation

In the genetic algorithm, the selection of crossover and mutation operators has a significant impact on the convergence of the algorithm [45]. The crossover operator is used primarily to generate new individuals and improve the search ability. The mutation operator can make the algorithm converge to the optimal solution and maintain the population diversity. In the running process of the algorithm, the crossover and mutation probabilities change according to the fitness value and no longer keep a value from start to end. To a certain extent, it overcomes the problem of the population falling into the local optimum and improves the convergence speed of the algorithm. The crossover and mutation probability are shown in Formulas (11) and (12).

The expression of adaptive crossover probability is as follows:

(11) $$p_{c}=\{\begin{array}{cc} \frac{\bar{f}-f^{\prime }}{\bar{f}-f_{\min}}\left(p_{c1}-p_{c2}\right)+p_{c2} & f^{\prime }\leq \bar{f}\\ p_{c2} & f^{\prime }>\bar{f} \end{array},$$

and the expression of adaptive mutation probability is:

(12) $$p_{m}=\{\begin{array}{cc} \frac{\bar{f}-f}{\bar{f}-f_{\min}}\left(p_{c1}-p_{c2}\right)+p_{c2} & f\leq \bar{f}\\ p_{m2} & f>\bar{f} \end{array}$$

where $p_{c1},p_{c2},p_{m1},p_{m2}$ are the upper and lower limits of the probability of crossover and mutation, respectively. In this paper, the values of each parameter are 0.8, 0.6, 0.05 and 0.01, respectively. $f^{\prime }$ is the smaller fitness value in the two crossed individuals. f is the fitness value of the mutant individual. $\bar{f}$ is the average fitness value of each generation.

It can be seen from Equations (11) and (12) that if the present individual has the smallest fitness value, the probability of cross mutation is the largest, and if the current individual fitness value exceeds the average fitness value of the population, the probability of cross mutation is the smallest. As a result, the diversity of the population will increase, and the probability of jumping out of the local optimal solution will increase as well.

In the later stages of the iteration of the standard genetic algorithm, the population is obliged to move closer to the optimal individual. The resulting consequence is necessary to reduce the diversity of the population and increase the risk of the algorithm falling into a locally optimal solution [46]. As a result, the immigration strategy has been applied, that is, the introduction of new individuals into the evolution process will increase the diversity of the population and thus improve the searchability of the algorithm.

To determine whether the conditions of the immigration strategy, one needs to calculate whether the diversity of the population has weakened. If the diversity of the population weakens, it means that the individuals retained by the algorithm tend to be equal, and the fitness function of the individuals is relatively close, so it is expected that the difference between the average fitness value of the individuals and the population will decrease. Let,

(13) $$F=\frac{1}{N}\sum_{j=1}^{N}\left|f_{i}-\bar{f}\right|$$

where $f_{i}$ is the fitness value of the i-th individual during the evolution of the population, $\bar{f}$ is the average fitness of the population and N is the number of the population. If F is lower than a certain value (the value is set to 0.024 in this paper), the default population is reduced and new chromosomes need to be introduced. Here new chromosomes are artificially added and immigration strategies are initiated.

**Figure 5:**
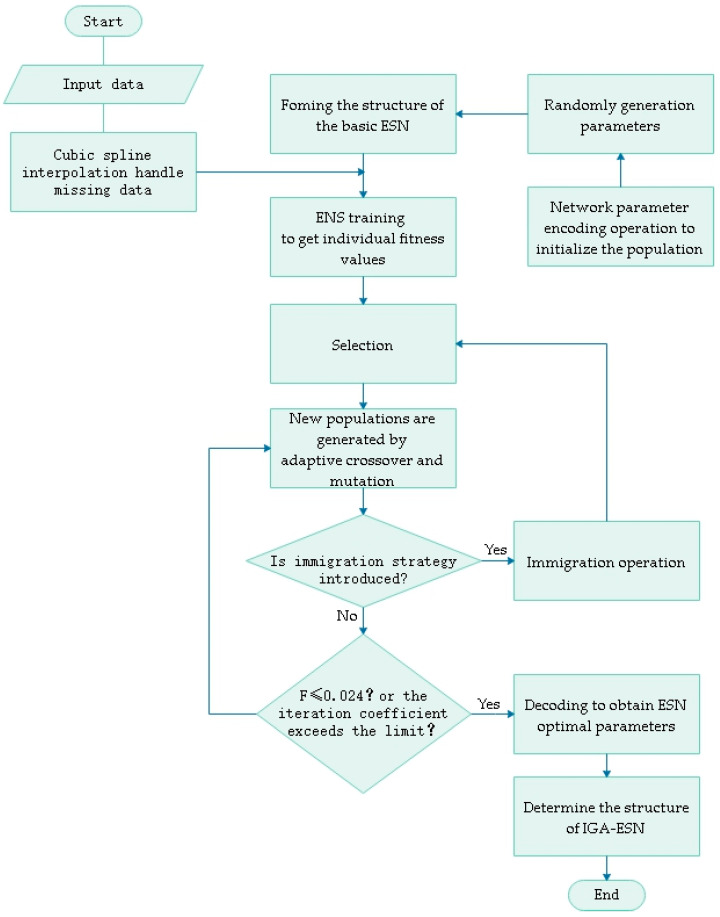
The development process of improved genetic algorithm optimization echo state network (IGA-ESN) soft sensor model.

To sum up, the development process of the IGA-ESN soft sensor model is summarized in Figure 5.

## 4. Experiment

In this study, to validate the feasibility and effectiveness of the IGA-ESN, the established soft sensor was applied to the estimation of alumina concentration in the operation process of an aluminum reduction cell in a modern aluminum plant. Then, to show the advantages of the soft sensor framework, the performance of the IGA-ESN model was compared to that of four other common DDM methods. For performance evaluation, the mean relative error (MRE) and root mean squared error (RMSE) indices were used.

### 4.1. Application Background

The modern large aluminum reduction cell is the core equipment of aluminum plants. It is a huge and complex high-temperature and high-current reactor under the interaction of multiple physical fields [47]. In practical application, it is connected in series and supplied by a low voltage direct current source.

**Figure 6:**
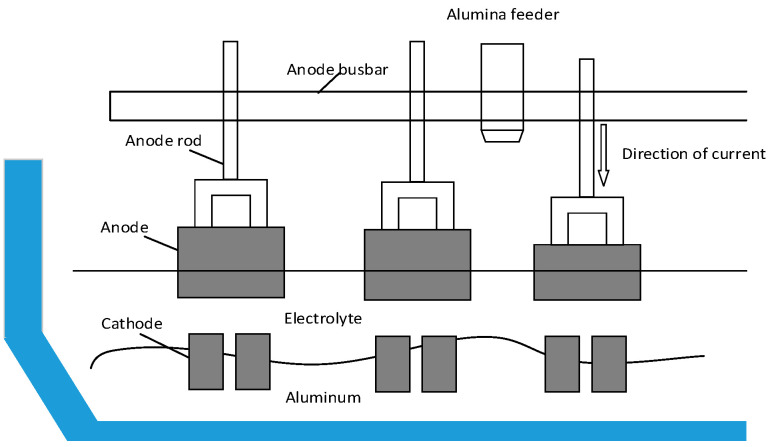
Internal structure of a typical reduction cell.

Commonly, a prebaked carbon anode cell structure (shown in Figure 2) is used. The anodes are sustained by anode rods, which are arranged in parallel on both sides. They are connected with an anode busbar on the corresponding side, and then form a parallel circuit with the common cathode. DC flows from anode to cathode, thus generating high temperature. During the electrolysis, alumina are added through the feeder at a certain time interval and dissolved into the electrolyte continuously to produce molten aluminum. Alumina in the electrolyte must be maintained in a certain amount for production to continue and in order to secure the stability, safety and efficiency of the cell. As a crucial process variable, alumina concentration would be required to stay within a required range. This is because excessive alumina can form “sludge”, which is sometimes difficult to dissolve and cause corrosion to the cathode. On the contrary, insufficient alumina will cause an anode effect, bringing about an abnormal sharp rise of cell voltage and an increase of greenhouse gas emissions [48]. Internal structure of a typical reduction cell is shown in Figure 6.

**Figure 7:**
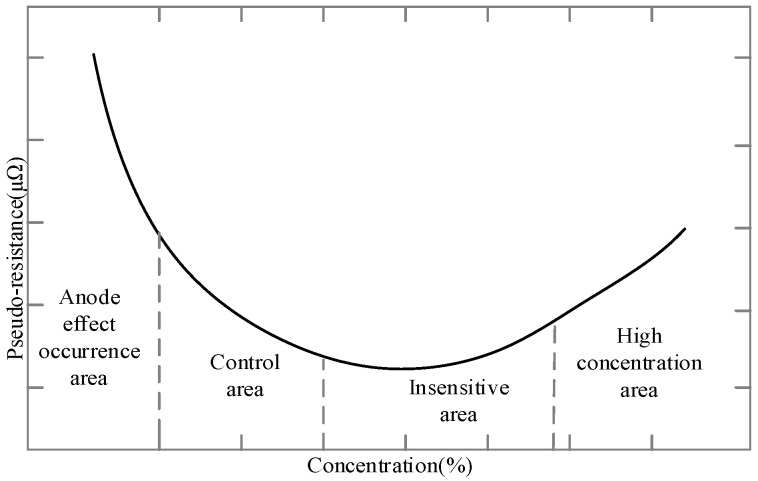
The relationship between cell pseudo-resistance and alumina concentration.

At present, alumina concentration control algorithm theory based on cell pseudo-resistance tracking is commonly used in the control system of the reduction cell. Depending on the relationship between cell pseudo-resistance and alumina concentration (namely the R–C curve), alumina concentration change is controlled by tracking the change of the cell’s pseudo-resistance signal. Based on the theoretical amount of alumina consumption, the alternate operation process of under feed and over feed is adopted, and alumina concentration in the electrolyte fluctuates near the optimal concentration point. The R–C curve is shown in Figure 7.

The above chart could consist of four areas: (1) High concentration area. The pseudo-resistance is highly sensitive to alumina concentration changes, but too high alumina concentration will cause precipitation at the bottom of the cell. (2) Insensitive area. The pseudo-resistance is extremely insensitive to changes in alumina concentration, and has a modest real-time response and low current efficiency. (3) Control area. The pseudo-resistance is more sensitive to alumina concentration changes, and the current efficiency is higher; this is the ideal control area for industrial alumina concentration. (4) Anode effect occurrence area. The mass of alumina is very low. As alumina concentration decreases, the resistance rises rapidly, and the anode effect phenomenon can easily occur.

The fluctuation range of alumina concentration should be controlled within a certain range. If the concentration is higher or lower than the concentration value corresponding to the outermost point of the cell pseudo-resistance, the cell pseudo-resistance will increase. In the process of aluminum reduction, the alumina concentration should appear in the control area as much as possible. By measuring the cell pseudo-resistance, according to the relationship between them, the purpose of indirectly measuring the alumina concentration is achieved.

In general, the cell pseudo-resistance can be obtained from an overall perspective:

(14) $$R_{i}=\frac{U_{i}-E}{I_{i}}$$

where $R_{i}$ is the cell pseudo-resistance at the i-th time, $U_{i}$ is the cell voltage at i-th time and $I_{i}$ is the line amperage of the cell at i-th time; normally, $I_{i}$ is approximately constant. E is the apparent back electromotive force.

### 4.2. Data Acquisition and Processing

Among the variables related to alumina concentration, in addition to cell voltage, the most easy to realize online real-time detection is the anode current distribution. We utilized the self-developed anode current distribution online detection device to obtain the required anode current distribution data. In addition, in order to assess the variables more accurately, the measurement of the voltage between anode and cathode was increased.

The detection principle of the anode current distribution is the equidistant voltage method. Two probes that keep a certain distance are set on the anode guide rod to form an equal distance voltage drop. By collecting the voltage at this distance, the current flowing through it is calculated according to Ohm’s law. The expression is as follows:

(15) $$I_{anode}=\frac{U_{anode}}{R_{anode}}$$

where $I_{anode}$ is the current flowing through each anode rod, $U_{anode}$ is the measured equidistant voltage of each anode rod and $R_{anode}$ is the equivalent resistance of the length between two points of the voltage sampling of the anode rod. During the aluminum reduction process, the temperature of the anode rod changes in real-time and this leads to changes in the resistivity of the anode rod. The actual resistance of the anode rod is:

(16) $$R_{anode}=\frac{\left(1+\alpha T\right)\rho_{Al}L}{S}$$

where α is the resistivity temperature coefficient, $\rho_{Al}$ is the resistivity of pure aluminum, L is the length between two points of the voltage sampling of the anode rod, S is the cross-sectional area of the anode rod and T is the temperature measured by the anode rod at the surface point.

**Figure 8:**
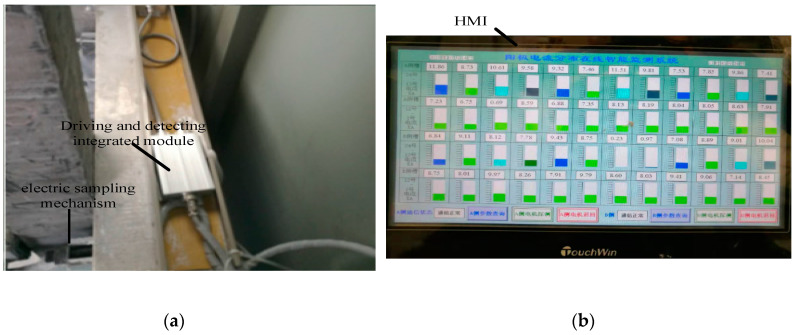
On-line anode current distribution detect device: (**a**) multichannel acquisition module; (**b**) human machine interface (HMI).

As shown in Figure 8, this self-developed device consists of an electric sampling mechanism, a driving and detecting integrated module, and an human machine interface (HMI) that has touch screen control and data visualization functions. It is light in weight, small in volume, anti-magnetic and high temperature and vibration resistant.

**Figure 9:**
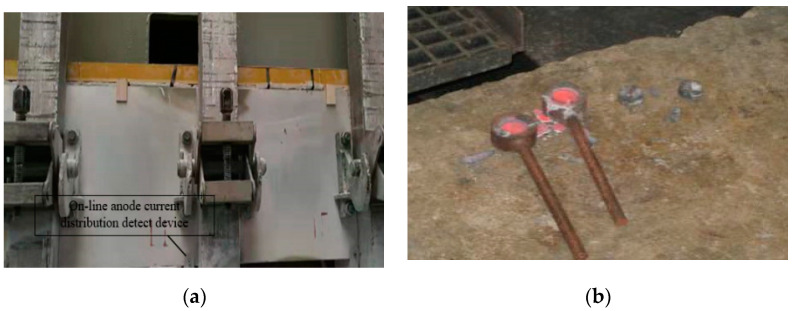
In situ data collection: (**a**) online collection of anode current distribution in aluminum reduction cell; (**b**) alumina concentration sampling for inspection.

The anode distribution data and the voltage between anode and cathode were synchronously acquired through the online monitoring terminal. At the same time, the data of alumina concentration were manually collected and measure offline in the laboratory. Finally, a total of 150 sets of valid data was achieved by benchmarking the anode current, bipolar voltage and offline alumina concentration. The collection device is shown in Figure 9.

In the process of collecting data, due to the harsh environment of the site, there will inevitably be missing data in the data collection process. This has a significant impact on the establishment of prediction models, so it is necessary to fill in missing data. The lost parts of the collected data are shown in Table 1.

Considering that the alumina concentration data are a time series data type with small short-term fluctuations, we selected the cubic spline interpolation method to deal with the missing data. The core operation of the cubic spline interpolation method is tantamount to construct a polynomial to form a smooth curve. This curve can fit the main data points very well, and the formed curve has higher convergence and further stability. Therefore, it applies well to time series processing [49].

The missing data in the collected data are shown in Table 1, followed by the cubic spline interpolation supplements in Table 2.

The results showed that the processed data were roughly consistent with the original data, and that there were no significant fluctuations. The basic characteristics of the data did not change, indicating that the interpolation method was effective. This accords with the principle of the interpolation method, which is beneficial to further research later.

### 4.3. Experimental Results of the Soft Sensor Model Based on IGA-ESN

In checking the feasibility of the method, 150 groups of valid data were collected to form a test sample set to verify the soft sensor model. The test sample set was split into two subsets. First 100 groups of data were used as the training subset of the model, and the last 50 groups of data were used to test the prediction performance of the model. The measured value of alumina concentration obtained from the sampling test was compared with the output of the soft sensor model to obtain the estimation performance evaluation of the model.

The mean relative error (MRE) and the root mean square error (RMSE) were used as the evaluation criteria. The MRE calculation formula is as follows:

(17) $$MRE=\sum_{i=1}^{N}\frac{\left|y_{i}-y\right|}{y}\times 100\%$$

and the RMSE calculation formula is:

(18) $$RMSE=\sqrt{\frac{1}{N}\sum_{i=1}^{N}{\left(y_{i}-y\right)}^{2}}$$

where N is the number of test samples, y is the real value and $y_{i}$ is the model output value.

**Figure 10:**
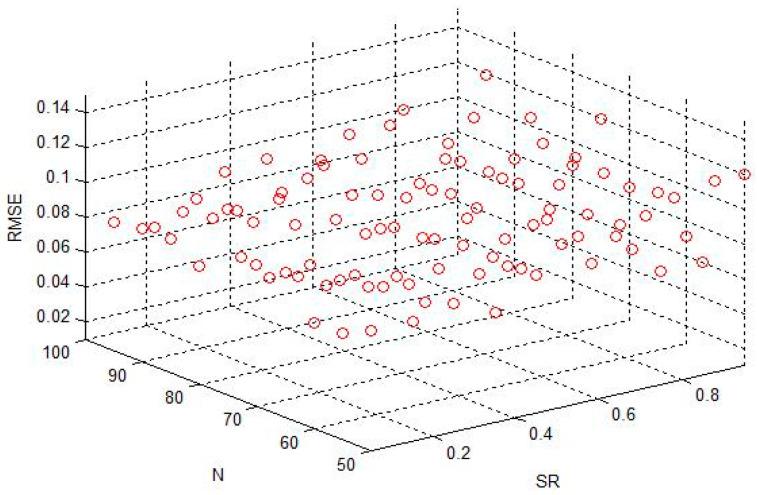
Standard deviation the root mean square error (RMSE) when spectral radius SR and reservoir size N change.

The variation range of SR was [0.05, 0.95], the change step size was 0.1, the transformation range of N was between [50, 95], the transformation step size was 5, IS = 0.2 and SD = 0.35t the average standard deviation output of the predicted alumina concentration is shown in Figure 10.

It can be seen from Figure 10 that with the input scale and the sparseness of the reservoir determined, as the spectral radius and the size of the reservoir changed, the standard deviation obtained by the training continuously changed.

**Figure 11:**
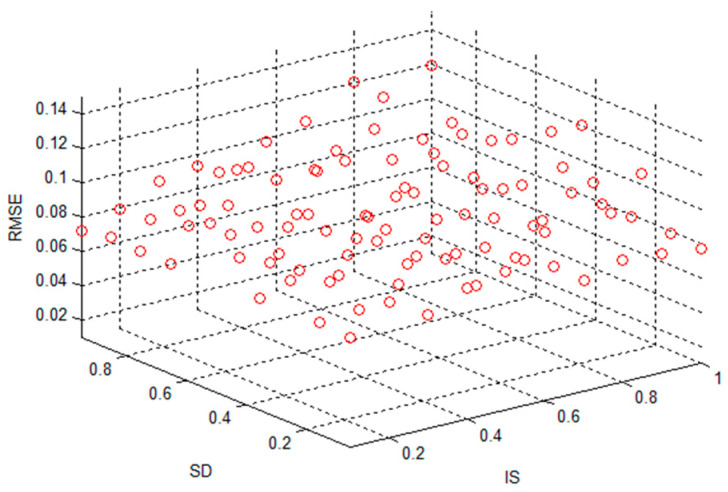
Standard deviation RMSE when input scale IS and reserve pool sparseness SD change.

The spectral radius SR was taken as 0.6, the size of the reserve pool was N = 80, IS = [0.1, 1], the step size was 0.1, SD = [0.05, 0.95], the step size was 0.1 and the average standard for the prediction of alumina concentration was obtained. The difference output is shown in Figure 11.

As can be seen from Figure 10 and Figure 11, different combinations of SR, N, IS and SD will greatly affect the performance of the network, and it is particularly important to use genetic algorithms to obtain appropriate parameters.

**Figure 12:**
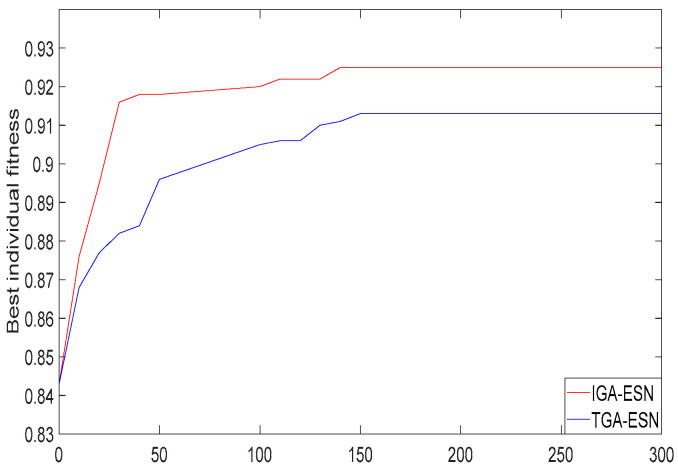
The best fitness curve of IGA-ESN.

Using the method of Section 3, the best fitness curve of the model is shown in Figure 12.

**Figure 13:**
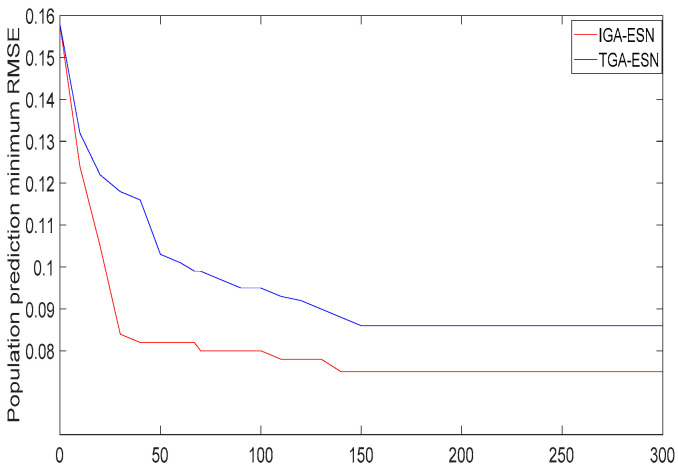
Minimum standard deviation curve of population prediction.

The minimum standard deviation of the predicted alumina concentration in population evolution can be calculated from Figure 12, as shown in Figure 13.

**Figure 14:**
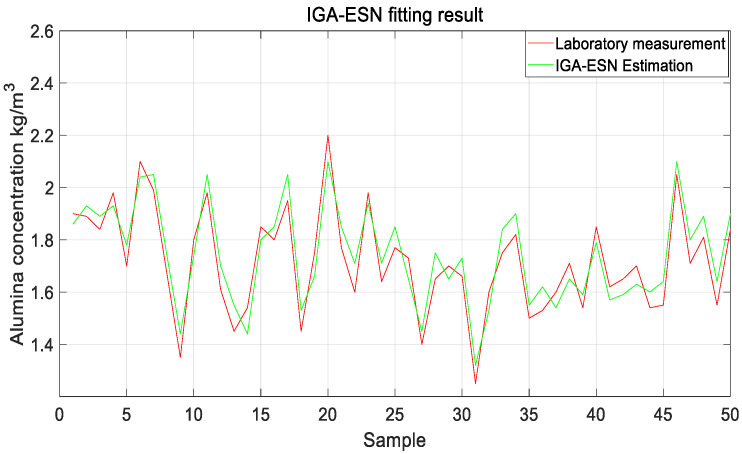
Fitting results of alumina concentration by IGA-ESN.

From Figure 13, it can be seen that IGA-ESN was faster than the individual optimization parameters of the ESN optimized by the traditional genetic algorithm (TGA-ESN); that is, the standard deviation of the model fitting decreased rapidly, indicating that the training speed was excellent and the IGA-ESN’s optimal parameters can be found faster. Additionally, it was easy to fit the production data. The prediction standard deviation of the model obtained by TGA-ESN was 0.0862, and the internal connection weight matrix SR of each parameter of TGA-ESN was calculated to be SR = 0.56, the size of the reservoir was N = 180, the scale of the input signal was IS = 0.43, and the degree of scarcity of the reservoir SD = 0.39. The model prediction standard deviation obtained by IGA-ESN was 0.0765, and the internal connection weight matrix of each parameter of the IGA-ESN decoding consisted of SR = 0.56, reservoir size N = 180, input signal scaling scale IS = 0.36 and reservoir scarcity SD = 0.43. The results of using IGA-ESN to predict alumina concentration in the subsequent 50 groups are shown in Figure 14. It can be seen that the optimized network prediction value was closer to the real value and the performance was better.

### 4.4. Comparison with Typical Methods

In this section, we compare the proposed method with typical data-driven methods, such as least-squares support vector machine (LSSVM), extreme learning machine (ELM), backpropagation (BP) and kernel extreme learning machine (KELM) and model-based methods, such as the multilayer state observer [50].

When the LSSVM algorithm is used for modeling, the kernel function uses a radial basis function kernel (RBF) and determines the approximate value range of parameters γ and $\sigma^{2}$ according to experience: $\gamma \in [\gamma_{\min},\gamma_{\max}],\sigma^{2}=[\sigma_{\min}^{2},\sigma_{\max}^{2}]$; seven values and five values of parameters γ and $\sigma^{2}$ are selected to form a 7 × 5 grid $\gamma =\left[1,2,5,10,20,30,50\right],\sigma^{2}=[0.05,0.1,0.5,1,5]$, forming 35 pairs of parameter combinations. Combined with 50 fold cross validation, the parameter combination of the minimum standard deviation RMSE was obtained; the penalty factor was γ=10 and the kernel function was $\sigma^{2}=1$. The input node of the ELM network was 2, the output was 1, the hidden layer node was set to 150, and the activation function was Sigmoid. The model input node of the BP network was 2, the output was 1, the hidden layer node was set to 150, and the activation function was Sigmoid. The KELM algorithm was also used to estimate alumina concentration (see reference [31] for details), and RBF was used as the kernel function. The standard deviation and average relative error obtained by training are shown in Table 3.

The RMSE and MRE of several training methods are shown in Table 3.

It can be seen from the comprehensive simulation results and model indicators that the simulation effect of the IGA-ESN model was better than the other algorithms’ simulation effect. For LSSVM, even if the optimal hyperparameters were obtained using grid search and cross-validation, the simulation effect maintained errors. From the simulation results, it can be noted that compared with the models established by LSSVM, BP, and ELM, the IGA-ESN had a standard deviation reduction of more than 39% and an average relative error of about 34%. It is concluded that in the aluminum production data, when the time series is used as the ESN model and the anode current and the voltage between anode and cathode are used to predict the alumina concentration, a reliable prediction effect can be obtained.

The model-driven approach and data-driven approach have their respective applicability. In reference [50], a multilayer state observer was proposed to estimate alumina concentration, and good results were obtained when the factory model was relatively accurate. The method divides the aluminum reduction trough into several interconnected subsystems according to the position of the feeder and assumes that other feeders are blocked, so as to estimate the alumina concentration of a subsystem more accurately. However, in cases where the plant model is not clear, the approach proposed in this paper is more effective. In general, if the application object is controlled independently by a single feeder and the physical model is more accurate, data-driven and model-based hybrid drive methods can be considered to estimate variations in local alumina concentration.

## 5. Conclusions

A soft sensor method based on an echo state network parameter model, IGA-ESN, was proposed. This method uses an improved genetic algorithm to optimize the key parameters of ESN, introduces an immigration strategy and adaptively changes the crossover and mutation frequency to improve the soft sensor accuracy. In order to verify the effectiveness of the method, we applied the method to the prediction of alumina concentration based on data collected in the production site of aluminum reduction. The results showed that the prediction of alumina concentration by this method was consistent with the actual sampling test value. Although the proposed method can realize the soft sensing of alumina concentration, the real-time performance and stability of the algorithm need to be further improved in practical applications. Therefore, the future work is to focus on the real-time practicability of the algorithm on the basis of ensuring measurement accuracy, so as to solve the dynamic real-time soft sensing problem. It is planned to integrate the proposed method and the developed monitoring system into the existing process control system, conduct real-time online calibration and optimize the structure, so as to improve the prediction accuracy and stability of the proposed method. Then, the data estimated by this method will be fed back to the process control system. In the planned method, after the actuator executes the control command, the state changes, and the contemporary estimation value is generated to the process control system. In this way, the method will be more real-time and practical, so as to realize the alumina concentration online soft sensor. In addition, we will also explore the application of this method to solve the multi-target soft sensor problems in the presence of multivariable coupling and uncertain interference.

## Figures and Tables

**Table 1:** Missing data.

Time	Al_2_O_3_ (kg/m^3^)	Voltage between Anode and Cathode (V)	Anode Current (kA)
15:29:31	2.23	3.809	6.459
15:29:47	2.42	3.794	6.547
15:30:01	3.12	3.794	6.457
15:30:14	2.96	3.799	6.453
15:30:30	2.77	3.809	6.366
15:30:46	2.92		6.277
15:31:00	2.68	3.813	6.099
15:31:15	3.18	3.809	6.275
15:31:29	3.22		6.45
15:31:44	3.29	3.804	6.532
15:31:59		3.789	6.272
15:32:23	3.45	3.794	6.273

**Table 2:** Data fitting by cubic spline interpolation.

Time	Al_2_O_3_ (kg/m^3^)	Voltage between Anode and Cathode (V)	Anode Current (kA)
15:29:31	2.23	3.809	6.459
15:29:47	2.42	3.794	6.547
15:30:01	3.12	3.794	6.457
15:30:14	2.96	3.799	6.453
15:30:30	2.77	3.809	6.366
15:30:46	2.92	3.812	6.277
15:31:00	2.68	3.813	6.099
15:31:15	3.18	3.809	6.275
15:31:29	3.22	3.809	6.45
15:31:44	3.29	3.804	6.532
15:31:59	3.22	3.789	6.272
15:32:23	3.45	3.794	6.273

**Table 3:** Comparison of experimental results of several models.

Model Type	RMSE	MRE (%)
LSSVM ^1^	0.136521	6.0953
ELM ^2^	0.127423	7.1056
BP ^3^	0.134296	6.8999
KELM ^4^	0.0832	4.7889
TGA-ESN ^5^	0.0862	4.8566
IGA-ESN ^6^	0.0765	4.5321

^1^ Least-squares support vector machine. ^2^ extreme learning machine. ^3^ Backpropagation. ^4^ Kernel extreme learning machine. ^5^ Optimization of echo state network by traditional genetic algorithm. ^6^ Optimization of echo state network by improved genetic algorithm.
